# Isolation of *Cronobacter *spp. (formerly *Enterobacter sakazakii) *from infant food, herbs and environmental samples and the subsequent identification and confirmation of the isolates using biochemical, chromogenic assays, PCR and 16S rRNA sequencing

**DOI:** 10.1186/1471-2180-9-225

**Published:** 2009-10-27

**Authors:** Ziad W Jaradat, Qotaiba O Ababneh, Ismail M Saadoun, Nawal A Samara, Abrar M Rashdan

**Affiliations:** 1 Department of Biotechnology and Genetic Engineering, Jordan University of Science and Technology, P. O. Box 3030, Irbid-22110, Jordan

## Abstract

**Background:**

*Cronobacter *spp. (formerly *Enterobacter sakazakii*), are a group of Gram-negative pathogens that have been implicated as causative agents of meningitis and necrotizing enterocolitis in infants. The pathogens are linked to infant formula; however, they have also been isolated from a wide range of foods and environmental samples.

**Results:**

In this study, 233 samples of food, infant formula and environment were screened for the presence of *Cronobacter *spp. in an attempt to find its source. Twenty nine strains were isolated from samples of spices, herbs, infant foods, and dust obtained from household vacuum cleaners. Among the 76 samples of infant food, infant formula, milk powder and non-milk dairy products tested, only one sample of infant food contained *Cronobacter *spp. (1.4%). The other *Cronobacter *spp. isolates recovered include two from household vacuum dust, and 26 from 67 samples of herbs and spices. Among the food categories analyzed, herbs and spices harbored the highest number of isolates, indicating plants as a possible reservoir of this pathogen. Initial screening with API 20E test strips yielded 42 presumptive isolates. Further characterization using 3 chromogenic media (α-MUG, DFI and EsPM) and 8 sets of PCR primers detecting ITS (internal transcribed spacer sequences), 16S rRNA, *zpx, gluA, gluB, OmpA *genes followed by nucleotide sequencing of some PCR amplicons did not confirm the identity of all the isolates as none of the methods proved to be free of both false positives or false negatives. The final confirmation step was done by 16S rRNA sequence analysis identifying only 29 of the 42 isolates as *Cronobacter *spp.

**Conclusion:**

Our studies showed that *Cronobacter *spp. are highly diverse and share many phenotypic traits with other *Enterobacteriaceae *members highlighting the need to use several methods to confirm the identity of this pathogen. None of the biochemical, chromogenic or PCR primers proved to be a reliable method for confirmation of the identity of the isolates as all of them gave either false positives or false negatives or both. It is therefore concluded that 16S rRNA sequencing is pivotal to confirm the identity of the isolates.

## Background

*Cronobacter *spp. (formerly *Enterobacter sakazakii*), a member of the *Enterobacteriaceae *family, are motile, non spore forming, Gram-negative facultative anaerobes. They are catalase positive, oxidase negative, and generally positive for α-D-glucosidase [1-4]. *Cronobacter *spp. have been repeatedly reported as remarkably resistant to osmotic stress and dryness and moderately thermotolerant as some encapsulated *Cronobacter *spp. were still recoverable from desiccated infant formula after storage for up to 2.5 years [5-7]. The composition of dry foods and infant formula combined with their low *aw *(ca. 0.2) significantly affected the survival of *Cronobacter *spp. in these foods [6,8,9].

*Cronobacter *spp. cause meningitis and necrotizing enterocolitis in infants, and septicemia and catheter-associated infections in elderly and immunocompromised people, with mortality rates ranging between 10 to 80% [10-17]. Among the cases, about half of the patients died within one week of the onset of the infections and about 94% of the meningitis survivors exhibited severe neurological complications [12,14,18].

Infant formula has been associated with severe systemic neonatal infections by *Cronobacter *spp., and thus these organisms are considered to be infant formula pathogens [11]. Nonetheless, *Cronobacter *spp. have been isolated from a wide range of habitats which include milk powder, formula constituents and from environments from within manufacturing plants [19-22], and household utensils such as blenders, infant bottle cleaning brushes and spoons [23-26]. Furthermore, they have been isolated from different types of foods such as rice, cured meat, sausages and minced meat, acidic sobia (a fermented beverage with pH range 3.4 -5.5), soured tea, lettuce, and other vegetables [27-31]. In humans, it has been isolated from cerebrospinal fluid, blood, skin wounds, breast abscess, urine, respiratory secretions and digestive tract samples [10,32,33]. In addition to food and clinical samples, *Cronobacter *spp. were isolated from various insect's intestinal tracts such as the Mexican fruit fly *Anastrepha ludens *and the stable fly *Stomoxys calcitrans*. They have also been isolated from rats, soil sediment, wetland, and even crude oil [34-39].

*Cronobacter *spp. was defined as a new species by Farmer et al. [19], before which, it was known as "yellow pigmented *Enterobacter cloacae." *It produces yellow pigmented colonies on trypticase soy agar (TSA), after 48-72 h [1]. However, the production of the yellow pigment is not exclusive for *Cronobacter *spp. as other organisms also produce yellow-colored colonies on TSA. In addition, it was found that not all *Cronobacter *spp. produces yellow color on TSA [2]. In a previous study, Farmer et al., [19] grouped 57 strains of *E. sakazakii *into 15 biogroups which later, Iversen et al., [40] expanded by using cluster analysis (based on partial 16S rRNA sequence analysis) of 189 strains to include a 16^th ^biogroup. This was followed by two proposals by Iversen et al. [41,42] showing that this organism comprised of six related groups of strains that could be separated on the basis of DNA-DNA hybridization relatedness and phenotypic traits, into 5 novel species and 1 novel genomospecies within a new genus named *Cronobacter*. These studies gave a clear indication of the genetic and phenotypic heterogeneity among these organisms. Therefore, it is important that the presence and the identity of *Cronobacter *spp. be confirmed by more than one method. Biochemical, chromogenic and molecular techniques such as PCR that amplify specific *Cronobacter *spp. genes and 16S rRNA sequencing analysis should be among the methods used for this purpose. The aims of this study therefore were to analyze a wide range of foods including infant foods, milk powder, herbs, and environmental samples in an attempt to find the reservoir for this pathogen and to compare the biochemical, cultural and molecular methods for the proper identification and confirmation of *Cronobacter *spp.

## Methods

### Samples collection

A total of 222 samples of food, infant formula, infant foods, herbs and spices originating from 14 different countries were purchased from local markets. In addition, 11 environmental samples (vacuum dust and soil) were collected and tested for the presence of *Cronobacter *spp.

### Isolation of *Cronobacter *spp

It is noteworthy to mention that in this study two methods of *Cronobacter *spp. isolation were used. The FDA method [43] was used at the beginning of the project for the isolation of *Cronobacter *spp. from the food and herbal samples. However, during the project, a new modified method for the isolation of *Cronobacter *spp. was developed [2]. Thus, the new method was adopted for the isolation of *Cronobacter *spp. from infant formula and milk powder samples.

### Isolation of *Cronobacter *spp. from infant formula, milk powder and infant foods

A total of 76 samples (40 infant formulas and solid infant foods, 29 milk powder and 7 dairy non-milk foods) were tested for the presence of *Cronobacter *spp. using the method described by Iversen and Forsythe, [2]. Briefly, 100 g of infant food, milk powder or infant formula were added to 900 ml of peptone water and warmed up for 25 min at 45°C. Ten milliliters were then incubated in *E. sakazakii *enrichment broth (ESE) for 24 h at 37°C. From each enriched sample, 0.1 ml and 1 ml were streaked or spread onto Druggan Forsythe Iversen (DFI, Oxoid, UK,) agar and incubated for 24 h at 37°C. Green/bluish colonies were considered presumptive *Cronobacter *spp. and were subjected to further biochemical and molecular confirmation techniques.

### Isolation of *Cronobacter *spp. from food, herbs andenvironmental samples

*Cronobacter *spp. were isolated from different food and herbal samples according to the FDA method [43] with modification. Briefly, 100 g of each sample were mixed thoroughly with 900 ml of pre-warmed sterile distilled water at 45°C, and incubated for 15-20 min in a water bath at the same temperature. Ten milliliters of each mixture were resuspended in 90 ml of *Enterobacteriaceae *enrichment broth (EE, HighMedia, India) and incubated overnight at 37°C. A loop full of the culture broth was streaked onto Violet Red Bile Glucose Agar (VRBGA, HighMedia, India) and another 0.1 ml of the same culture was spread onto VRBGA agar plates and incubated for 20-24 h at 37°C. All colonies were streaked onto tryptic soy agar (TSA) and incubated for 24-48 h at 37°C to look for the characteristic yellow colonies of *Cronobacter *spp. All colonies that appeared yellow on TSA were picked and subjected to further characterization using biochemical, chromogenic, PCR and 16S rRNA sequencing analysis. Confirmed cultures were preserved in EE broth containing 20% glycerol and stored at -80°C for further studies.

### Biochemical characterization by API 20E test strips

Presumptive identification of oxidase-negative yellow colonies was performed by API 20E (Remel and/or BioMerieux, USA) biochemical profiling test according to manufacturer's instructions.

### Chromogenic assays for environmental isolates

API 20E *Cronobacter *spp. positive isolates were streaked onto nutrient agar containing 4-methyl-umbelliferyl α-D-glucoside (α-MUG, Oxoid, UK,) a substrate which upon being metabolized forms yellow colonies that fluoresce under UV light. The same isolates were then further confirmed by streaking onto DFI chromogenic agar containing 5-bromo-4-chloro-3-indolyl-α, D-glucopyranoside (XαGlc, Oxoid, UK,) which upon hydrolysis of the substrate gives blue/green colonies typical for *Cronobacter *spp. Further, the presumptive isolates were inoculated onto the EsPM chromogenic medium (R & F Laboratories, Downers Grove, IL) on which typical *Cronobacter *spp. colonies appeared blue/black as described by Restaino et al. [21].

### Molecular confirmation of the isolates using PCR and sequencing

Eight sets of *Cronobacter *spp.-specific primers were used in the study and are listed in Table 1. Primers SG-F/SG-R and SI-F/SI-R, originally described by Liu et al. [44], were deduced from alignment of the internal transcribed spacer sequences. Primers Saka 1a -F/Saka 2b-R described by Hassan et al. [45] were deduced from variable region of the 16S rRNA gene. Primers ESSF/ESSR described by Nair and Venkitanarayanan [46] were deduced from the *OmpA *gene. Two primer sets reported by Kothary et al. [13] were deduced from the *zpx *gene. Lastly, PCR primers reported by Lehner et al. [3,47] were deduced from the α-glucosidase genes, *gluA *and *gluB *(ORFs 71, 72) sequences. Reactions using primers Saka1a-F/Saka2b-R and SG-F/SG-R and SI-F/SI-R and ESSF/ESSR were optimized in a 50 μl reaction mixture consisting of 5 μl of the bacterial genomic DNA solution (50 ng), 3 mM MgCl_2_, 0.25 μM (each) dATP, dCTP, dTTP and dGTP; 2 U Taq DNA polymerase, 1.25 μl (0.25 μM each) primers and 33.1 μl nuclease free water. PCR products were analyzed using 2% (w/v) agarose gel electrophoreses in 0.5 × TBE buffer and a constant voltage of 90 V to confirm the presence of amplified DNA. PCR assays using primers for *zpx *and *gluA/gluB *were according to parameters and conditions reported by the authors who originally described each PCR assay. For BAM primers (350 bp product), initially the PCR analysis was performed on all of the strains using reaction that used the 62°C annealing temperature. However, eight of the strains produced multiple bands in addition to the 350 bp amplicon. Gradient PCR analysis of these strains was performed to find the best annealing temperature that give only one band (unpublished data). From this analysis, an annealing temperature of 50.5°C was selected to complete the study. Surprisingly, the lower annealing temperature gave one band which upon DNA sequencing appeared to be the correct one while the other non-specific bands disappeared. This unexpected result might be due to the use of the Invitrogen Platinum PCR super mix that was used at 50.5°C but not at other temperatures.

**Table 1:** Oligonucleotide primer pairs and PCR running conditions used in this study

Primer	Sequence 5' to 3'	Targeted site	Amplicon size (bp)	Reference
SG-F	GGGTTGTCTGCGAAAGCGAA^a^	ITS-G	282	Liu et al., [44]
SG-R	GTCTTCGTGCTGCGAGTTTG	ITS-G & ITS-IA		
				
SI-F	CAGGAGTTGAAGAGGTTTAACT^b^	ITS-IA	251	Liu et al., [44]
SI-R	GTGCTGCGAGTTTGAGAGACTC	ITS-G & ITS-IA		
				
Saka 1a	ACAGGGAGCAGCTTGCTGC^c^	V1^g^	952	Hassan et al., [45]
Saka 2b	TCCCGCATCTCTGCAGGA	V3^h^		
				
Zpx F	GAAAGCGTATAAGCGCGATTC^d^	*zpx*	94	Kothary et al., [13]
Zpx R	GTTCCAGAAGGCGTTCTGGT			
				
BAM122	AWATCTATGACGCGCAGAACCG^e^	*zpx*	350	Kothary et al., [13]
BAM123	AAAATAGATAAGCCCGGCTTCG			
				
EsgluAf	TGAAAGCAATCGACAAGAAG^f^	*gluA*	1680	Lehner et al., [3]
EsgluAr	ACTCATTACCCCTCCTGATG			
				
EsgluBf	TGAGTGAAGCACCGACGCAG^f^	*gluB*	1720	Lehner et al., [47]
EsgluBr	GTTACGTCACAGGTTTTGAT			
				
ESSF	GGATTTAACCGTGAACTTTTCC^i^	*ompA*	469	Nair and Venkitanarayanan [46]
ESSR	CGCCAGCGATGTTAGAAGA			

^a^&^b ^Running conditions; 94°C for 10 min; 30 cycles of 94°C for 30 sec each; 57°C for 1 min; 72°C for 1 min; a final extension period of 5 min at 72°C.

^c ^Running conditions; 95°C for 4 min; 30 cycles of 95°C for 60 sec each; 50°C for 1 min; 72°C for 90 sec; final extension period of 4 min at 72°C.

^d^&^e ^Running conditions; The hot start polymerase was activated by incubation for 15 min at 95°C; followed by 35 cycles of 1 min at 95°C; 62°C for *zpx *primers (50.5°C was used for 8 isolates) or 50°C for BAM primers for 1 min; 72°C for 1 min; final extension of 10 min at 72°C for *zpx *and 7 min for BAM primers.

^f ^Running conditions were as described by Lehner et al. [3,47]; The hot start polymerase was activated by incubation for 15 min at 95°C; followed by 30 cycles of 30 s at 94°C; 56°C (*gluA*) or 58°C (*gluB*) for 1 min; 72°C for 1.5 min; final extension period of 5 min at 72°C.

^g^&^h: ^Variable regions of the 16S rRNA gene.

^i ^Running conditions: 94°C for 2 min; 30 cycles 94°C for 15 sec each; 60°C for 15 sec; 72°C for 30 sec; final extension period of 5 min at 72°C.

### DNA sequencing

All products for nucleotide sequencing including the desalted PCR amplicons were obtained by using a QIAquick PCR Purification Kit according to the manufacturers' instructions (Qiagen). The questionable 400 bp amplicons obtained from the BAM degenerate PCR primers, were sequenced utilizing Amersham Biosciences ET Terminator chemistry using an ABI 377 DNA sequencer (Amplicon Express).

### 16S rRNA sequencing

DNA sequencing for the 16S rRNA segment was performed as described by Iversen et al. [41]. PCR amplification of the ribosomal RNA gene was performed by mixing 1 μl of extracted DNA with a 49 μl of PCR mixture containing the following: 1× GeneAmp PCR buffer, 5 units AmpliTaq Gold DNA polymerase (Applied Biosystems), 0.2 mM dNTPs, 1.5 mM MgCl_2 _and 1 pmol from primers P0 (5'-AGA GTT TGA TCC TGG CTC AG-3') and P6 (5'-GTA CGG CTA CCT TGT TAC GA-3'). PCR amplification was performed as follows: 10 min at 95°C; 30 cycles of 30 sec at 95°C, 30 sec at 56°C, 2 min at 72°C; 5 min at 72°C. The amplified products were visualized on 1% agarose gels, and then they were cut out from the gel and purified using the Wizard SV Gel and PCR clean-up system (Promega). The purified amplified fragments were sequenced using the primers P6 (5'-GTA CGG CTA CCT TGT TAC GA-3'), 095P (5'-TAC GGC GTG GAC TAC CAG-3') and the BigDye Termination Kit (Applied Biosystems). Full-length 16S rRNA gene sequences were aligned and compared with the DNA sequences deposited in the GenBank by Iversen et al. [41] using alignment tool MegAlign of the DNAStar program package.

### Submission of 16S rRNA gene sequences

All the obtained 16S rRNA gene sequences were submitted to the GenBank. The accession numbers of these sequences are listed in Table 2.

**Table 2:** *Cronobacter *spp. isolates and the Genbank accession numbers of their 16S rRNA sequences.

Isolate number	GenBank accession number	Isolate number	GenBank accession number
146A_095P.seq	FJ906897	175_095P. seq	FJ906898
s20B.seq	FJ906899	22_095P.seq	FJ906900
s32.seq	FJ906901	s44A.seq	FJ906902
s44B.seq	FJ906903	s52.seq	FJ906904
s77.seq	FJ906905	s93.seq	FJ906906
s95.seq	FJ906907	s96.seq	FJ906908
s112.seq	FJ906909	s146B.seq	FJ906910
s148.seq	FJ906911	s149.seq	FJ906912
s154.seq	FJ906913	s160A.seq	FJ906914
s160B.seq	FJ906915	s170.seq	FJ906916
s171.seq	FJ906917	s172.seq	FJ906918
s173.seq	FJ906919	s174.seq	FJ906920
ss176.seq	FJ906921	s178.seq	FJ906922
ss183.seq	FJ906923	s184.seq	FJ906924
s204.seq	FJ906925		

## Results and Discussion

This investigation addresses the isolation of *Cronobacter *spp. from a wide range of foods and environmental samples in an attempt to pinpoint their source. Because of the phenotypic differences among *Cronobacter *spp., it has been increasingly difficult to confirm the identity of isolates using only one method or one set of *Cronobacter *spp.-specific PCR primers [33,48]. Thus, this study also addresses the use of different chromogenic, biochemical, and molecular techniques for characterization and identification of *Cronobacter *spp. from foods and environmental samples.

Two hundred and thirty three samples including infant formulas, dry milk powder, infant foods, vegetables, fruits, traditional drinks, cereals, herbs, and environmental samples were tested for the presence of *Cronobacter *spp. Table 3 shows the categories of food and environmental samples analyzed for the presence of *Cronobacter *spp. in the study. Table 3 also indicates the percentages of *Cronobacter *spp. found in each food category, while Table 4 shows the description of foods, beverages and environmental samples which were positive for *Cronobacter *spp. Among the 76 samples of infant formula, infant food, milk powder and dairy non-milk food products, only one infant food sample was positive for *Cronobacter *spp. (1.4%). The highest percentage of *Cronobacter *spp. isolates (39%) was found in herbs and spices and totaled about 89.6% of the total isolates in this study. In addition, two isolates (18%) were recovered from vacuum dust collected from house holds. It is worth mentioning, that none of the tested milk powder samples contained *Cronobacter *spp. These results are in accordance with those described by Iversen and Forsythe [49], and Nazarowec-White and Farber [4] who suggested that pasteurization treatment when used in the final treatment stage eliminates all pathogens from such products. In contrast, other foods and beverages contained the highest levels of *Cronobacter *spp. For instance, the four samples of a traditional herbal drink, (liquorice) contained *Cronobacter *spp. (100%) while 11 out of 15 samples (73.3%) of mixed spices contained *Cronobacter *spp. These results are in accordance with reported results by Forsythe [11] and Friedemann [31] which emphasized that the majority of *Cronobacter *spp. isolates are from plant sources irrespective of the world region of analysis. These results imply that plants possibly embody the major reservoir of the pathogen.

**Table 3:** Categories of food and environmental samples tested for the presence of *Cronobacter *spp. and the numbers and percentages of the confirmed *Cronobacter *spp. isolates

Origin of Sample	Number of samples analyzed	Number of *Cronobacter *spp. isolates	% of total samples in the category	% of total isolates
Infant formula and milk powder	69	1	1.4	3.5
Cereals and Cereal	32	0	0	0
products				
Herbs and Spices	67	26	39	89.6
Miscellaneous food	34	0	0	0
products				
Beverages	7	0	0	0
Dairy products other than milk powder	7	0	0	0
Vegetables and fruits	6	0	0	0
Environmental samples	11	2	18.2	6.9

**Total**	**233**	**29**		**100**

**Table 4:** Detailed description and percentages of food, beverages and environmental samples which contained *Cronobacter *spp. isolates

Sample Type	Number of Samples of a category	Number of *Cronobacter *spp. isolates	% of samples positive for *Cronobacter *spp.
**Infant Formula and infant Foods**			
Infant foods	40	1	2.5
**Herbs and Herbal Beverages**			
Liquorice	4	4	100
Thyme	4	1	25
Anise	8	4	50
Chamomile	8	2	25
Fennel	6	3	50
Sage	2	1	50
**Mixed Spices**	15	11	73.3
**Environmental (vacuum dust)**	6	2	33.3

**Total**	**93**	**29**	**31.2**

Whether the *Cronobacter *spp. contamination is occurring intrinsically, i.e., endophytically or through contact with water, rodents, soil or insects during the primary preparation of these food products [11,18] has yet to be determined. Apparently, *Cronobacter *spp. survives the primary processing, shipping and exportation procedures well due to its thermo/dry/osmotic tolerant nature. Therefore, our results along with those previously reported, further confirm that *Cronobacter *spp. are ubiquitous microbes found in a wide array of foods and beverages including infant formula. However, due to its thermotolerant [7] and osmotolerant nature [6], the organism survives in dry foods, herbs, spices and the general manufacturing environment and appears to contaminate infant formula and infant foods at certain stages during the processing, particularly after sterilization i.e., during a vitamin or supplement fortification steps.

Nevertheless, previous studies by Shaker at al. [22] and Mullane et al. [16] reported conflicting results, in that, the former study reported a lack of *Cronobacter *spp. from 40 samples taken from an infant food factory, while the latter study lasting 12 months, isolated approximately 80 *Cronobacter *spp. isolates from infant food factories. Of these isolates, 72.5% were isolated from the factory environment. These findings provide evidence for the role of the environment in the contamination of the final product. It is interesting to note that in the current study, two *Cronobacter *spp. isolates were found in house-hold vacuum dust. This further supports the hypothesis of the role played by environmental contamination in factories or during the formula preparation in nurseries or the homes [31]. The high association of this pathogen with herbs and spices suggests that extra precautions should be taken when home remedies containing herbs or herbal beverages are given to infants to alleviate gastrointestinal discomfort. It should be mentioned that our findings reflect possibly an underestimation of *Cronobacter *spp. which might be associated with the foods (other than infant formula, infant food and milk powder) and environmental samples analyzed by the FDA BAM method because of only working up "yellow-pigmented colonies". However, these findings also support the need of isolation schemes that incorporate multiple chromogenic media.

All isolates were evaluated for phenotypic traits using biochemical profiling test (API 20E), and chromogenic (α-MUG, DFI and EsPM) assays while evaluated for genotypic traits using PCR analyses (eight sets of primers) and 16S rRNA sequencing. According to the initial screening, 56 isolates showed yellow colonies on TSA, typical for *Cronobacter *spp. However, when the isolates were subjected to API 20E biochemical profiling, only 42 isolates (75%) were identified as *E. sakazakii *with high identity scores (80-99% *E. sakazakii*) (Tables 5 and 6) and thus were considered presumptive *Cronobacter *spp. API 20E biochemical profiling can thus be considered a first screening or presumptive identification method for *Cronobacter *spp., after which the isolates should undergo further diagnostic analyses. To that end, the presumptive isolates were grown on chromogenic media (α-MUG, DFI and EsPM) as a second step of identification. Results showed that none of the three chromogenic media was 100% reliable (Table 7) for confirming the identity of *Cronobacter *spp. isolates. However, it is worth mentioning that both chromogenic α-MUG and DFI gave no false negatives and only few false positives (5 and 3 for α-MUG and DFI respectively) compared to the EsPM media which missed 3 positives and identified 7 non-*Cronobacter *spp. isolates as *Cronobacter *spp. These results proved that DFI followed by α-MUG are more reliable than the EsPM Media as intermediate confirmation steps. Among the non-*Cronobacter *spp. isolates, two isolates did not grow on DFI media although they tested positive for α-glucosidase activity on α-MUG. These isolates may be sensitive to the sodium deoxycholate, an ingredient added to the medium to suppress gram positive bacteria [1].

**Table 5:** Confirmed isolates of *Cronobacter *spp. by biochemical testing (API 20E), chromogenic (α-MUG, DFI and EsPM), eight sets of *Cronobacter *spp. specific primers (α-*GluA*, α-*GluB*, SG, SI, Saka, *OmpA*, *zpx *and BAM) and 16S rRNA sequence analysis.

Isolate						PCR Primers								
						
ID	Source	API 20E	α-MUG	DFI	EsPM^$^	α-GluA	α-GluB	SG	SI	Saka	OmpA	zpx	BAM^€^	16S rRNA
51329	ATCC	+	+	+	BB	+	ND^§^	+	+	+	+	+	+*	*Crono.*^£^
29544	ATCC	+	+	+	BB	+	+	ND	ND	ND	ND	+	+	*Crono.*
Jor32	Infant food	+	+	+	BB	+	ND	+	+	+	+	+	+	*Crono.*
Jor20B	Spices	+	+	+	BB	+	ND	+	+	+	+	+	+	*Crono.*
Jor22	Chamomile	+	+	+	BB	+	ND	+	+	+	+	+	+	*Crono.*
Jor44A	Spices	+	+	+	BB	+	ND	+	+	+	+	-	+	*Crono.*
Jor44B	Spices	+	+	+	BB	+	ND	+	+	+	+	+	+	*Crono.*
Jor77	Anise	+	+	+	BB	+	ND	+	+	+	+	+	+*	*Crono.*
Jor93	spices	+	+	+	BB	+	ND	+	+	+	+	-	+	*Crono.*
Jor95	Anise	+	+	+	BB	+	ND	+	+	+	+	+	+*	*Crono.*
Jor96	Fennel	+	+	+	BB	+	ND	+	+	+	+	-	+	*Crono.*
Jor112	Liquorice	+	+	+	BB	+	ND	+	+	+	+	+	+*	*Crono.*
Jor146B	Liquorice	+	+	+	BB	+	ND	+	+	+	+	-	+	*Crono.*
Jor148	Spices	+	+	+	BB	+	ND	+	+	+	+	+	+	*Crono.*
Jor149	Anise	+	+	+	BB	-	-	+	+	+	+	+	+*	*Crono.*
Jor154	Spices	+	+	+	BB	-	+	+	-	+	+	+	+	*Crono.*
Jor160A	Vac dust^¥^	+	+	+	BB	+	ND	+	+	+	+	+	+	*Crono.*
Jor160B	Vac dust^¥^	+	+	+	BB	+	ND	+	+	+	+	-	+	*Crono.*
Jor171	Fennel	+	+	+	BB	+	ND	+	+	+	+	+	+*	*Crono.*
Jor172	Spices	+	+	+	BB	+	ND	+	+	-	+	+	+	*Crono.*
Jor173	Spices	+	+	+	BB	+	ND	+	+	+	+	+	+	*Crono.*
Jor174	Anise	+	+	+	BB	+	ND	+	+	+	+	-	+*	*Crono.*
Jor175	Spices	+	+	+	BB	-	-	+	+	+	+	+	+	*Crono.*
Jor176	Thyme	+	+	+	BB	+	ND	+	+	-	-	-	+*	*Crono.*
Jor183	Spices	+	+	+	BB	+	ND	+	+	+	+	+	+	*Crono.*
Jor204	Liquorice	+	+	+	BB	+	-	+	+	+	+	+	+	*Crono.*
Jor146A	Liquorice	+	+	+	BB	+	ND	+	+	+	+	+	+	*Crono.*
Jor178	Chamomile	+	+	+	BB	+	ND	+	+	+	+	-	+	*Crono.*
Jor52	Sage	+	+	+	Y/Gr	-	ND	-	-	-	-	-	-*#	*Crono.*
Jor170	Fennel	+	+	+	Gray	-	ND	-	-	-	+	-	-	*Crono.*
Jor184	Spices	+	+	+	Y/Gr^##^	-	ND	+	+	+	+	+	-	*Crono.*

**Total +**		**31**	**31**	**31**	**28**	**25**	**2**	**28**	**27**	**26**	**28**	**21**	**28**	

$On EsPM, colonies were blue black (BB) in chromogenic reaction color within 24 h at 37°C. **€**The PCR conditions for BAM primers as described in Table 1 were used for amplification of both regions of the *zpx *gene as described by Kothary et al. [13]. Analysis of the *Cronobacter *and non-*Cronobacter *strains was performed in a similar fashion. ¥ Vacuum dust. ND§: not determined. * Multiple bands. *#, PCR product was approximately (400 bp) and sequence was found not to be *zpx*. ##Colonies were blue black (BB) after three days at 37°C. *£ Crono; Cronobacter *spp.

**Table 6:** Presumptive *Cronobacter *spp. as appeared through testing the isolates by biochemical profiling (API20E), chromogenic (α-MUG, DFI, EsPM) and eight sets of *Cronobacter *spp- specific primers (α-*gluA*, α-*gluB*, SG, SI, Saka, *OmpA*, *zpx *and BAM), while confirmed as non-*Cronobacter *spp. by 16S rRNA sequence analysis.

Isolate						PCR Primers								
						
ID	Source	API 20E	α-MUG	DFI	EsP M	α-GluA	α-GluB	SG	SI	Saka	OmpA	zpx	BAM^€^	16S rRNA
Jor20A	Spices	+	-	-	Clear	-	ND	+	+	-	-	+	-	N.*Crono*
Jor27	Chamomile	+	-	-	Y^&^	-	ND	+	+	-	-	+	-	N.*Crono*
Jor45	Sugar	+	-	-	Gray	-	ND	+	+	-	-	+	-	N.*Crono*
Jor115A	Dates	+	+	NG^@^	Y/Gr	-	ND	-	-	-	-	+	-	N.*Crono*
Jor115B	Dates	+	+	NG^@^	Y/Gr	-	ND	-	-	-	-	+	-*#	N.*Crono*
Jor51	Dry dairy	+	+	+	Y/Gr##	-	ND	+	+	-	-	+	- #	N.*Crono*
Jor153B	Semolina	+	+	+	BB	-	-	+	+	-	-	+	-	N.*Crono*
Jor26	Rice	+	-	-	BB	-	-	+	+	-	-	+	+	N.*Crono*
Jor100	Semolina	+	-	-	BB	+	ND	+	+	-	-	+	+	N.*Crono*
Jor103	Spices	+	-	-	BB	+	ND	+	+	-	-	+	+	N.*Crono*
Jor109	Grapes	+	-	-	BB	+	ND	+	+	-	-	+	+	N.*Crono*
Jor168	Spices	+	-	-	BB	-	-	+	+	-	-	+	+	N.*Crono*
Jor151	Fennel	+	+	+	BB	-	+	-	-	-	-	+	+	N.*Crono*

**Total +**		**13**	**5**	**3**	**7**	**3**	**1**	**10**	**10**	**0**	**0**	**13**	**6**	

**€**The PCR conditions for BAM primers as described in Table 1 were used for amplification of both regions of the *zpx *gene as described by Kothary et al. [13]. * multiple bands. *#, PCR product was approximately (400 bp) and sequence was found not to be *zpx*. & Y, yellow colony chromogenic reaction color, 24 h at 37°C. Gr, green colony chromogenic reaction color, 24 h at 37°C. @ NG; no growth on DFI at 37°C. ##Colonies were blue black (BB) after three days at 37°C. N. *Corono*; None *Cronobacter *spp.

**Table 7:** Summary of the performance of the biochemical, chromogenic and PCR methods for *Cronobacter *spp. identity confirmation.

Test Method	Number of confirmed isolates	Number of false positives	Number of false negatives
**API 20E**	42	13	-
**α-MUG**	34	5	-
**DFI**	32	3	-
**EsPM**	33	7	3
**Glu A PCR primers**	26	3	6
Glu B*PCR primers	3	1	3
**SG PCR primers**	36	10	2
**SI PCR primers**	36	10	3
**Ska PCR primers**	25	-	4
**OmpA PCR primers**	27	-	2
**Zpx 94 bp PCR primers**	32	13	10
**Zpx 350 bp PCR primers**	32	6	3

*: Only 9 samples were tested using these primers

Surprisingly, the identities of 13 of the isolates (Table 6) identified as *E. sakazakii *by API 20E analysis were not confirmed by the other methods used including chromogenic, PCR and the final 16S rRNA sequence analysis. There have been several comparative studies performed to determine the usefulness of biochemical test strips and chromogenic as a diagnostic tool for the identification of *Cronobacter *spp. However, these studies have given conflicting results [48,50,51] highlighting the need for other methods of confirmation such as molecular and the DNA sequencing methods.

PCR analysis using eight different sets of primers from six separate studies [3,13,44-47] was used to help ascertain the identity of all the presumptive isolates. Standard ATCC strains (51329 and 29544) were used as a positive control. Although eight sets of PCR primers from six different studies each claiming high sensitivity and specificity for detection and confirmation of *Cronobacter *spp. were used to ascertain the identity of the isolates in this study, only 13 isolates in addition to the ATCC (51329) strain were positive with all the primers (Table 5). The other 16 isolates did not give the predicted PCR product with at least one set of primers although they were identified as *Cronobacter *spp. by other biochemical and/or chromogenic methods. When the isolates were tested with the PCR primer sets, DNA was not amplified in a high number of strains especially when tested with the *zpx *(94 bp product) and *gluB *detecting only 21/31 and 2/5 respectively. The other sets of primers where more reliable detecting 25/31, 26/30, 27/30, 28/31 for *gluA*, Saka, SI and BAM primer sets respectively while both *OmpA *and SG appeared to be most reliable among the tested primer sets detecting 28/30 isolates.

These observations suggest that there may be some sequence variability in the genes of these strains of *Cronobacter *spp. that were not observed by the reporting authors [3,13,47].

In addition, it is noteworthy to mention that strains Jor149, Jor154, Jor175, Jor 52, Jor170, Jor184, Jor51, Jor153B and Jor151 gave conflicting α-glucosidase activity (on α-MUG or DFI) that did not correspond with PCR results for the presence of *gluA*. All these strains had expressed α-glucosidase activity on both α-MUG and DFI, but were negative by PCR for the presence of *gluA*. Because of these results we tested some of the *gluA *PCR negative strains with primers that targeted *gluB *by using primers, parameters and PCR reaction conditions described by Lehner et al [47]. The PCR results showed that these primers did not detect *gluB *in the tested strains (except strains Jor 151 and Jor 154), suggesting that the α-glucosidase activity observed was due to the expression of a second uncharacterized α-glucosidase gene or that these strains possessed *gluA *and *gluB *sequence variants that were not recognized by these primers under the reported conditions. These results are in contrast with results from strains Jor151 and Jor154 which also had α-glucosidase activity, but PCR results showed that *gluB *was present and not *gluA*, suggesting that the glucosidase activity of these strains was due solely to the expression of *gluB*. These results are also opposite to that for strain Jor204 which by PCR showed that its α-glucosidase activity was due to *gluA *and not *gluB*. Lastly, Strains Jor 26, Jor 100, Jor 103, Jor 109, and Jor168 expressed no α-glucosidase activity during growth on α-MUG or DFI yet produced typical chromogenic reactions on EsPM suggesting that these strain's chromogenic activity on the latter medium was due to cellobiosidase activity or due to expression of sequence variants of α-glucosidase genes. The later outcome is the most probable reason for these conflicting results since all of these strains showed either α-glucosidase activity or palatinase activity by VITEK GN analysis (data not shown). These results support the finding by Iversen and Forsythe [2] that the chromogenic reaction of strains grown on DFI medium can be misleading and that the new modified formulation of DFIA put forth by Iversen et al [48] should alleviate the problems of strains producing atypical reactions on this medium.

Table 6, depicts the characterization of the non *Cronobacter *spp. isolates. All the isolates were identified as putative *Cronobacter *spp. with API 20E biochemical profiling. However, chromogenic media (α-MUG and DFI) were negative for 8 isolates (Jor20A, Jor27, Jor45, Jor26 Jor100, Jor103, Jor109, and Jor168) while positive for the other 5 isolates (Jor115A, Jor115B, Jor51, Jor151 and Jor153B) and EsPM was negative for 6 samples and positive for 7 samples. These conflicting results stressed the inability of chromogenic methods to provide a reliable test for confirming the identity of the *Cronobacter *spp. isolates. Table 7 summarized the results obtained by the different methods used for the identification and confirmation of isolates and clearly highlights the inability of any single method to be used as a final confirmation method. Due to the above conflicting results, a final confirmation step was undertaken by sequencing the 16S rRNA gene of the isolates. As a result of final confirmation method only 29 isolates (Table 5) were confirmed as *Cronobacter *spp. while the other 13 isolates (Table 6) were confirmed as non-*Cronobacter *spp.

The variation in the above results reflects the genetic heterogeneity among the *Cronobacter *spp. isolates and/or a high degree of similarity between *Cronobacter *spp. and some other closely related members of *Enterobacteriaceae *that tested positive with some of the confirmation tests as depicted in Table 6. This conclusion is supported by very recent reports by Iversen et al., [41,42] and Barron et al., [33] who have proposed a new scheme for classifying *E. sakazakii *isolates based on f-AFLP, DNA-DNA hybridization, riboprinting and full-length, 16S rRNA gene sequences and phenotypic characteristics.

## Conclusion

*Cronobacter *spp. are ubiquitous in nature, and herbs and spices appear to be one possible natural reservoir and thus special care should be taken while preparing infant foods or formulas in order to avoid cross-contamination from these sources. Finally, the *Cronobacter *spp. are very diverse as indicated by the variation in the confirmation results both phenotypic and genotypic. Among the methods, the α-MUG and DFI could be used for putative identification of *Cronobacter *spp. followed by the SG, *OmpA *and BAM PCR analysis. However, the 16S rRNA sequence analysis should be used as a final confirmation step and is pivotal for eliminating the doubts shed by the inability of other methods for identification and confirmation of the identity of the *Cronobacter *spp. Therefore, a combination of confirmation methods might be necessary to completely eliminate false positives and false negatives.

## Authors' contributions

ZWJ wrote the proposal for the fund, supervised all the experimental work and wrote the manuscript. QOA participated in the PCR experiments, 16S rDNA sequencing and alignment, and manuscript writing. IMS participated in supervising the work at the laboratory. NAS isolated the *Cronobacter *spp. isolates from foods. AMR participated in PCR experiments and chromogenic identification of the pathogens. All authors read and approved the final manuscript.
